# Fungal Endophyte Communities of Crucifer Crops Are Seasonally Dynamic and Structured by Plant Identity, Plant Tissue and Environmental Factors

**DOI:** 10.3389/fmicb.2020.01519

**Published:** 2020-07-15

**Authors:** Junhui Chen, Komivi Senyo Akutse, Hafiz Sohaib Ahmed Saqib, Xiaolu Wu, Feiying Yang, Xiaofeng Xia, Liande Wang, Mark S. Goettel, Minsheng You, Geoff M. Gurr

**Affiliations:** ^1^State Key Laboratory of Ecological Pest Control of Fujian and Taiwan Crops, College of Plant Protection, Fujian Agriculture and Forestry University, Fuzhou, China; ^2^Institute of Applied Ecology, Fujian Agriculture and Forestry University, Fuzhou, China; ^3^Joint International Research Laboratory of Ecological Pest Control, Ministry of Education, Fuzhou, China; ^4^International Centre of Insect Physiology and Ecology, Nairobi, Kenya; ^5^Graham Centre, Charles Sturt University, Orange, NSW, Australia

**Keywords:** Brassicaceae, colonization rate, land use, endophytism, symbiont, biodiversity

## Abstract

Endophytic fungi are important in diverse plant functions but knowledge of the factors that shape assemblages of these symbionts is lacking. Here, using a culture-dependent approach, we report 4,178 endophytic fungal isolates representing 16 orders isolated from stems, roots and leaves of three cruciferous plant species, Chinese cabbage (*Brassica rapa* L.), radish (*Raphanus sativus* L.) and white cabbage (*B. olerocea* L.), collected from 21 focal fields with different landscape contexts and pesticide uses during four seasons (summer, autumn, winter and spring). The colonization rate of fungi was found to be most strongly affected by season, plant identity and plant tissue. The colonization was highest during autumn, followed by summer, spring and lowest during winter. The colonization was highest in *B. olerocea* (53.2%), followed by *B. rapa* (42.6%), and lowest in *R. sativus* (35.0%). The colonization was highest in stems (51.9%) in all plant types, followed by leaves (42.4%) and roots (37.5%). Hypocreales was the dominant order (33.3% of all the isolates), followed by Glomerellales (26.5%), Eurotiales (12.1%), Pleosporales (9.8%) and Capnodiales (6.0%). Fungal endophyte abundance (number of isolates) followed the same pattern as colonization rate, while species richness varied with season and host plant tissue. Ordination analyses showed that the abundance and richness of Hypocreales, Eurotiales and Sordariales were associated with plant roots, while Capnodiales, Pleosporales and Trichosphaeriales were associated with spring. Other environmental factors, elevation, and the proportions of grassland, forest, orchard and waterbodies in the surrounding landscape also exerted effects within some categories of other main effects or for certain fungal taxa. Our results indicate that while fungal endophyte communities of crucifer crops vary strongly with the season, they are also strongly structured by plant identity and plant tissue, to a lesser extent by pesticide use and only weakly by landscape composition. The understanding of the ecological roles of fungal endophytes could contribute to habitat management and consequently improve crop pest management.

## Introduction

Fungal endophytes have fundamental roles in plant ecology and physiology such as plant growth regulators, increasing tolerance to biotic and abiotic stresses and as mediators of plant-insect and plant-soil interactions (Carroll, 1988; Berg, 2009; Vega et al., 2009; Almario et al., 2017; Shikano et al., 2017). Community structure and distribution patterns of endophytes in host plants are influenced by a variety of factors including agricultural practice, genetics of host plants, geography, season and vegetation structure in the surrounding landscape (Guo et al., 2008; Gasser and Berg, 2011; Nair and Padmavathy, 2014; Fort et al., 2016; Alzarhani et al., 2019; Sridhar, 2019). Understanding the ecological role of fungal endophytes could help design habitat management tools leading to improve pest management in crops.

Endophytes can have a mutualistic, symbiotic or neutral relationship with host plants, depending on the identity of the host plant (Redman et al., 2001; Unterseher and Schnittler, 2010; Kia et al., 2017). The pattern of colonization and distribution of endophytic fungi can vary between host plant species (Saikkonen et al., 2004; Peršoh, 2013, 2015). The spatial distribution is known to vary between roots, stems and leaves of the host plant (Rajagopal et al., 2010; Ghimire et al., 2011; Tong et al., 2011; Zheng et al., 2016). In one study examining *Brassica napus*, 97 endophytic fungi belonging to 40 species were identified but where they occurred varied with 35 from roots, 49 from stems and 13 from leaves. Furthermore, two of these endophyte species appeared in all parts of the plant, eight in two parts of the plant and 30 only in one part (Zhang et al., 2014). The colonization of specific plant tissues is based on their utilization of specific substrate that occur in that plant part (Rodrigues, 1994), while some endophytes can colonize the whole plant (Sun et al., 2018).

The community structure of microorganisms can vary between organic or conventional farming practices (Mäder et al., 2000; Clifton et al., 2015). Organic farming, in which synthetic chemical inputs such as pesticides are avoided, can reduce the detrimental impacts of conventional farming, by increasing biodiversity and reducing pesticide residues (Bengtsson et al., 2005; Reganold and Wachter, 2016). Microorganisms, similar to a number of other organisms including arthropods, are generally more abundant and diverse in organic farms (Bengtsson et al., 2005; Hole et al., 2005; Crowder et al., 2010; Gasser and Berg, 2011; Tuck et al., 2014; Ramos et al., 2017). Landscape structure and composition also plays a key role in the dynamics of microbial communities and interactions with their plant or arthropod hosts. Soil-borne endophytes can be beneficial to plants via growth promotion and induced resistance, which could influence herbivore performance. Moreover, the emission of volatile organic compounds as attractants to beneficial insects may change according to microbe-plant interactions (Pineda et al., 2010).

The distribution and growth of endophytes also vary across seasons and elevation gradients (Gao et al., 2005; Guo et al., 2008; de Mesquita et al., 2018; Ni et al., 2018). Elevation gradients and seasons are associated not only with the differences in the temperature, relative humidity and precipitation but also shifts in the vegetation and soils (Hashizume et al., 2008; Rojas-Jimenez et al., 2016; Siddique and Unterseher, 2016).

To date, many studies examining diversity patterns of endophytes have focused on factors such as agricultural practice, vegetation and elevation for pharmaceutical plants and other crops (Bengtsson et al., 2005; Gao et al., 2005; Meyling and Eilenberg, 2007; Guo et al., 2008; Hashizume et al., 2008; Mishra et al., 2012; Peršoh, 2013; Zhang et al., 2014; Rojas-Jimenez et al., 2016; Ramos et al., 2017). Trees or *Microthlaspi* plants are examples of highly studied endophytic communities and the factors that influence them (Zimmerman and Vitousek, 2012; Bálint et al., 2015; Eusemann et al., 2016; Glynou et al., 2016). However, very little is known about the relative strength of these factors and how the interactions shape the cruciferous vegetable endophyte communities. Accordingly, we hypothesized that endophytic fungal communities in non-mycorrhizal cruciferous vegetables are structured by a series of factors including landscape, season, farming practices, plant identity, plant part and elevation. The objectives of this study were to investigate the colonization of endophytic fungal community in non-mycorrhizal cruciferous vegetables and how multiple factors, landscape, farming practices, season and elevation shape the fungal endophyte communities.

## Materials and Methods

### Study Area, Land Use Survey and Sampling

**FIGURE 1:**
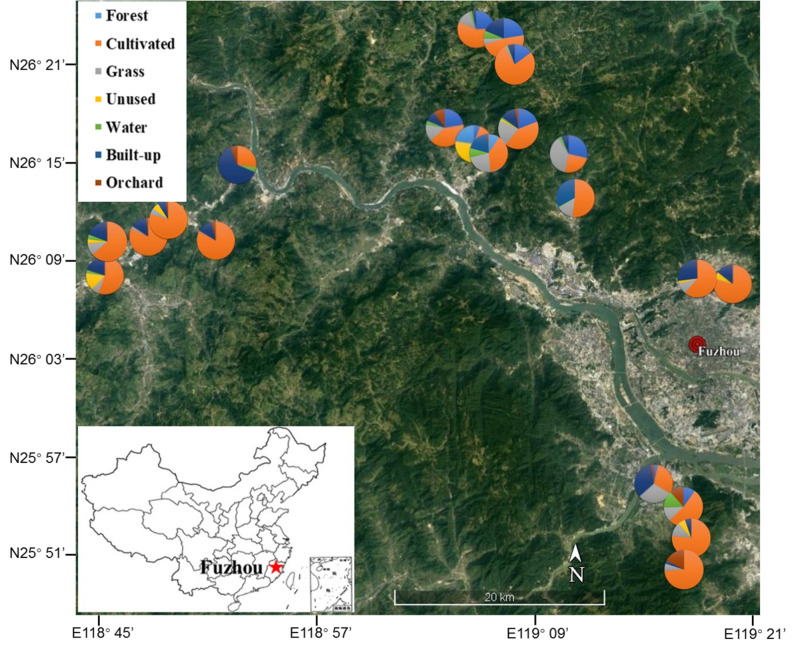
Location of focal vegetable fields in the region of Fuzhou City, Fujian Province, China. Pies show the composition of the landscape at 130 m radius around focal fields. Image obtained from Google satellite map (https://maps.google.com/) in January 2019.

Vegetable fields in Fujian Province, southeastern China (N25.55°– N26.20°, E118.43°– E119.31°) were selected to represent the range of organic and conventional (pesticide-sprayed) farms and surrounding landscapes. The area of each of the 21 fields was approximately 1,300–2,000 m^2^ and the distance between each field was at least 1 km (Figure 1). Three non-mycorrhizal cruciferous plant species, Chinese cabbage (*Brassica rapa* L.), radish (*Raphanus sativus* L.) and white cabbage (*Brassica olerocea* L.), were represented across both organic and conventional fields. Longitude, latitude and elevation were recorded by GPS (GPSMAP^®^ 60CSx-Garmin). The proportion of different land uses around the focal fields was quantified at a spatial scale of 130 m radius using the pictures taken by drone camera (PHANTOM 4, Shenzhen Dajiang Baiwang Technology Co., Ltd., China) and QGIS (version-2.18.27) tools. The land uses were classified into 7 categories: forest (naturally grown trees), cultivated land (tilled land, annual and perennial crops), grassland (artificial grassland, natural grassland, hedgerows and shrubs), unused land (barren land with little to no vegetation), water (rivers, ponds, irrigation channels and reservoirs), built-up (e.g., roads, residential land, greenhouses and factories) and orchard (e.g., loquat, litchi and citrus) (Supplementary Figure S1) (see Supplementary Table S1 for complete details of focal sites) (Zou et al., 2017).

In total, 71 sampling events took place during spring, summer, autumn and winter between July 2016 to April 2017. Cruciferous vegetables are planted in every season in Fujian region. The crop diversity in this arable land was high because farmers’ fields are small and dispersed. Samples were collected from all the selected fields at least once per season, but at times cruciferous plants in some sites were not found (see Supplementary Table S1 for complete details of sampling events). From each selected crop field, one or two of the three crop species was collected, based on the diversity of crops present. For each crop species, ten symptomless healthy-looking cruciferous plants were randomly collected in different lanes within the field in each season. The whole plants were uprooted and placed individually into polybags, labeled and stored in an ice cool box, transported to the laboratory and processed within 24 h.

### Plant Surface Sterilization and Fungal Endophyte Isolation

Samples were thoroughly washed in running tap water. Each sampled plant was cut into three parts: root, stem and leaf. Each root or stem was sectioned into 5 pieces (about 5 mm in length); each leaf was sectioned randomly to acquire 5 squares (around 20 mm × 20 mm). Although sample size influenced the abundance of isolates recovered, it had no influence on species richness (Gamboa et al., 2003). Therefore, we standardized the section sizes of these cruciferous plants. Sections were surface-sterilized by immersing in 75% ethanol for 2 min, transferred into 1.5% sodium hypochlorite for 3 min, and followed by three washes in sterilized distilled water under aseptic conditions (Hallmann et al., 2007). The surface sterilized pieces were placed onto sterilized filter papers until the residual water evaporated and then transferred onto potato dextrose agar (PDA; Oxoid) medium supplemented with 0.1% chloramphenicol. The effectiveness of the surface sterilization was tested by pipetting 100 μl from the last rinsed distilled water onto PDA medium. The PDA plates with the plant materials were incubated at 25 ± 1°C in complete darkness. Fungal outgrowth was checked at 4, 7, 10, 15, and 30 days and fungal colonization was assessed in each isolated plant tissue. Actively growing fungi were isolated and sub-cultured onto new PDA plates for purification. Several sub-cultures were conducted to acquire pure fungal isolates prior to their identification.

### Fungal Identification

Fungal identification was based on morphological and molecular methods. We classified fungi into different groups based on their morphological characteristics (colony appearance, pigmentation on both the top and reverse plates, growth rate, etc.); where isolates were grouped morphologically and only representative isolates from these groups were used for sequencing (molecular identification) (Hibbett et al., 2016). The explicit operation contained two phases, DNA extraction and polymerase chain reaction (PCR) amplification. Three DNA extraction methods were used: either repetitive freeze-thawing (Manian et al., 2001), urea solution (Sansinforiano et al., 1998) or CTAB (Posadas et al., 2012). The urea solution method was found most effective and used for the majority of isolates. Primer pairs targeting the internal transcribed spacer (ITS) regions of the rDNA, including ITS-5-F (5′-GGAAGTAAAAGTCGTAACAAGG-3′) and ITS-4-R (5′-TCCTCCGCTTATTGATATGC-3′), were used to amplify ∼672 bp fragment of fungal genome (White et al., 1990). PCR mixture contained 2 μl of template DNA, 1 μl (10 mM) from each forward and reverse primers, 12.5 μl of 2 × Phanta Max Buffer, 0.5 μl of dNTP Mix (10 mM each), 0.5 μl of Phanta Max Super-Fidelity DNA Polymerase (Vazyme biotech Co., Ltd., Nanjing, China), and 7.5 μl RNase free ddH_2_O to make a total volume of 25 μl. PCR cycling conditions were as follows: a pre-denaturation at 95°C for 5 min, 35 amplification cycles (denaturation at 95°C for 30 s, annealing at 59°C for 30 s, elongation at 72°C for 45 s), and a final extension at 72°C for 10 min. Gel electrophoresis with 1.0% agarose was performed to test the success of target DNA fragment amplification. PCR products were kept at −4°C before being sent to BioSune (Shanghai) for sequencing. The sequencing results were blasted in the GenBank database at the National Center for Biotechnology Information (NCBI^1^) after sequence editing. In total, 372 sequences were acquired. The standard of the blasted results depended on an identical percentage (>97%) and 110 operational taxonomic units (OTUs) were acquired eventually. The representative ITS sequences were submitted to GenBank and assigned serial numbers MN202660 to MN202769^2^ (see Supplementary Table S2 for complete details).

### Data Analysis

Fungal natural colonization rate was calculated by dividing the number of pieces exhibiting fungal outgrowth by the total number of pieces plated (Fröhlich et al., 2000). Negative binomial regression was used to test the significant variations of colonization rate, species richness and abundance (number of isolates) between categorical variables including season, plant identity, plant tissue and pesticide use (Mangiafico, 2016). Pearson correlation tests were conducted to detect the association of the top 10 fungal endophyte orders’ abundance and species richness with environmental variables (plant tissue, season, plant identity, pesticide use, and landscape composition) based on correlation. To avoid the abuse of correlation analyses in microbial ecology (Carr et al., 2019), associated p-values were adjusted to control the false discovery in multiple correlations (Benjamini and Hochberg, 1995). Fungal endophyte species census data was Hellinger transformed before multivariate ordination analysis. Hellinger transformation made the use of Euclidean distance-based ordination methods possible because the fungal endophyte data contained many zeros (absence of specific fungal endophytes in certain fields) (Legendre and Gallagher, 2001). We calculated species richness and abundance for the top 10 fungal endophyte orders. We chose the species richness in each order rather than selecting the overall fungal endophytes richness because different orders have different functional roles in the ecosystem. Based on species richness and abundance, community matrices were then analyzed using the “vegan” package (Oksanen et al., 2019) in R software.

We pooled the data based on plant tissues. Five tissues (e.g., roots) were pooled into as one plant tissue sample; ten plant tissue samples were pooled into one site sample, so we got three pooled data (roots, stems and leaves) for every sampling event. Canonical correspondence analysis (CCA) was conducted to detect variance explained by environmental variables in the community structure of the top 10 endophyte orders in terms of both species richness and abundance (Ter Braak, 1986; Ter Braak and Verdonschot, 1995). Variance inflation factors of all environmental variables in CCA models were calculated to test their collinearity with each other. The environmental variables which had high collinearity with other environmental variables (variance inflation factor value is higher than 10) were removed from final CCA models (Belsley et al., 1980). Thus, two variables (proportions from the built-up and orchard landscapes) were removed from the final CCA models. In addition, a permutation test was conducted to test the significance of CCA models and environmental predictors (Legendre and Gallagher, 2001). Ordination plots were generated to visualize the associations of fungal communities with environmental predictors. All calculations and analyses were conducted in R, using “vegan” (Oksanen et al., 2019) and “gplots” (Warnes et al., 2016) packages.

## Results

### Composition of Fungal Endophytes

**FIGURE 2:**
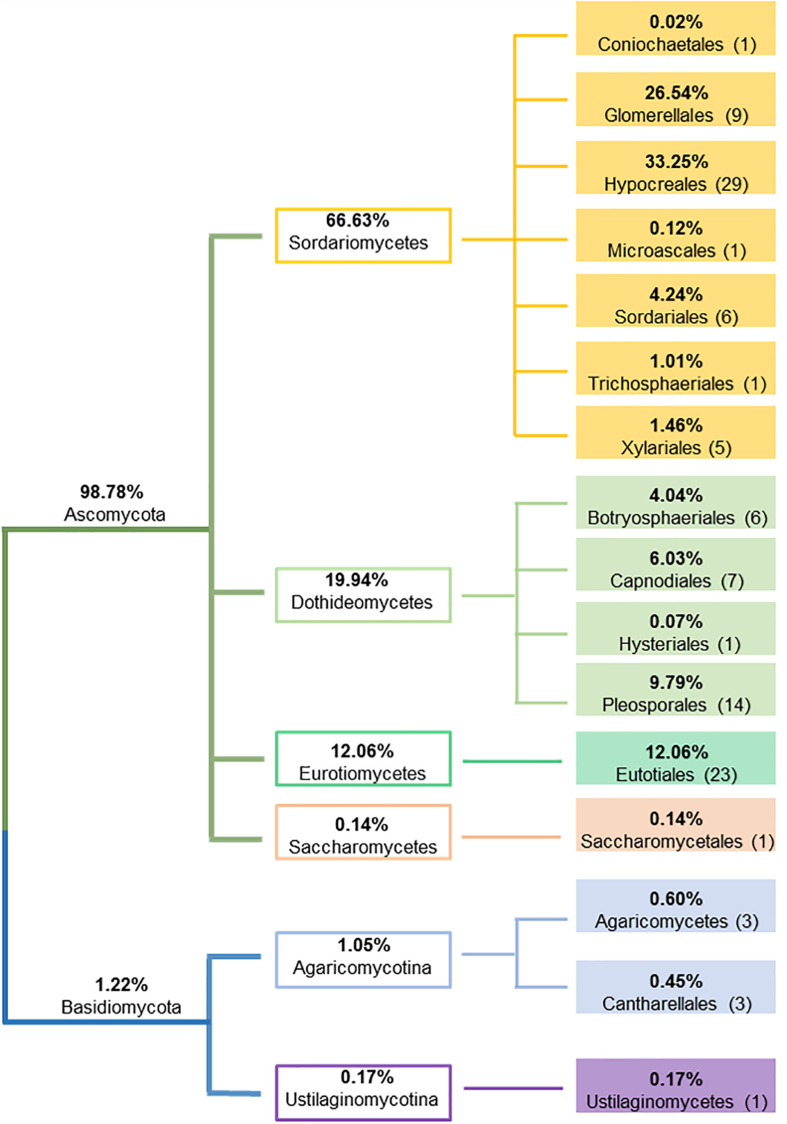
Schematic representation of the relative proportion of fungal endophytes at phylum, class and order levels.

A total of 4,178 endophytic fungal isolates, representing 2 phyla, 6 classes, 16 orders, 30 families, 51 genera and 110 species were isolated from 9,289 plant roots, stems and leaves (see Supplementary Table S3 for complete species information). Some plant segments were colonized by two or more isolates. Hence the number of fungal isolates colonized plant segments were lower (3,959) than the total fungal isolates, with an overall colonization rate of 43%. Ascomycota was the dominant fungal phylum consisting of 104 species (with 98.8% of overall representation), followed by Basidiomycota having only 6 species (with 1.2% of overall representation). In Ascomycota, the most dominant taxa were Sordariomycetes (Hypocreales, Glomerellales and Sordariales), Eurotiomycetes (Eurotiales), and Dothideomycetes (Pleosporales, Capnodiales and Botryosphaeriales), while Agaricomycotina represented the majority of fungal taxa in Basidiomycota (Figure 2). The most dominant endophyte genus was *Colletotrichum* (26.4% of total isolates), followed by *Fusarium* (24.8%), *Cladosporium* (5.9%) and *Trichoderma* (5.4%). Out of 110 totally detected species, 91 were isolated from leaves, 91 from stems, and 81 from roots; where 64 endophytic fungal species were shared by all plant tissues. Ten endophytic fungal species (*Aspergillus bombycis*, *Aspergillus nomius*, *Aspergillus penicillioides*, *Aspergillus tamarii*, *Aspergillus westerdijkiae*, *Cladosporium subuliforme*, *Cladosporium xanthochromaticum*, *Trichoderma hamatum*, *T. harzianum* and *Zopfiella* sp.) were isolated from stems only, ten (*Alternaria alternata*, *Alternaria burnsii*, *Phialemoniopsis pluriloculosa*, *Aspergillus flavipes*, *Colletotrichum siamense*, *F. fujikuroi*, *Gilmaniella subornata*, *Macrophomina* sp., *Mycotribulus mirabilis* and *Penicillium citrioviride*) from leaves only, and only three (*Beauveria bassiana*, *Coniochaeta* sp. and *F. equiseti*) from roots, whereas all other species were detected in at least two plant tissues. No fungal colonies grew from the last rinsed water plates, indicating the efficacy of the surface sterilization procedure and confirming that the fungi growing from the surface sterilized plant materials were endophytes originated from within the host plant tissues.

### Differences in Endophyte Colonization Rate, Species Richness and Abundance Between Season and Local Drivers

**FIGURE 3:**
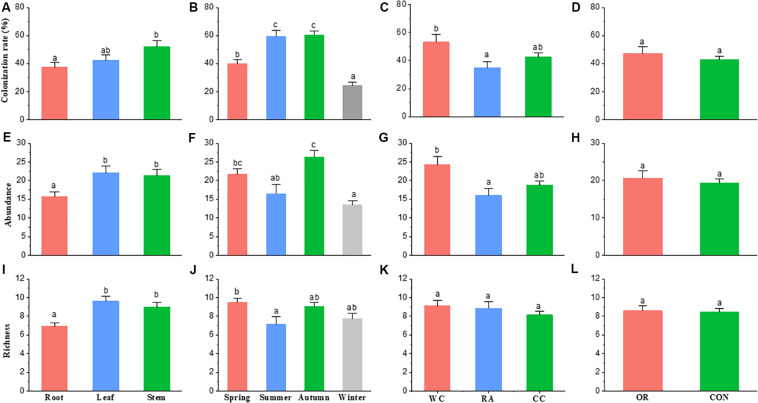
The difference in fungal endophyte colonization rate, abundance and richness within [**(A)**, **(E)**, and **(I)** respectively] host plant parts, [**(B)**, **(F)**, and **(J)** respectively] seasons, [**(C)**, **(G)**, and **(K)** respectively] host plant species and [**(D)**, **(H)**, and **(L)** respectively] farming practice. The bar shows the mean + standard error; same letters on the bars are not significantly different at level of *P* < 0.05. CC, Chinese cabbage; WC, white cabbage; RA, radish; OR, organic; CON, conventional farm.

Season (χ^2^ = 66.337, *df* = 3, *P* < 0.001), plant identity (χ^2^ = 7.3068, *df* = 2, *P* = 0.026) and plant tissue (χ^2^ = 7.0986, *df* = 2, *P* = 0.029) were drivers to the colonization rate of fungal endophyte communities. However, pesticide use was not (χ^2^ = 0.6789, *df* = 1, *P* = 0.41). Endophyte colonization rate in autumn and summer were higher than in spring and winter. White cabbage had a higher colonization rate of endophytes than radish (*P* = 0.022). The colonization rate of stem fungal endophytes was higher than the root fungal endophytes (*P* = 0.024). Organic and conventional farms (i.e., pesticide use) showed no difference in fungal endophyte colonization rate (Figures 3A–D).

Season (χ^2^ = 36.125, *df* = 3, *P* < 0.001), plant identity (χ^2^ = 9.2408, *df* = 2, *P* = 0.010) and plant tissue (χ^2^ = 10.295, *df* = 2, *P* = 0.006) were drivers to the abundance of fungal endophyte communities, but not pesticide use (χ^2^ = 0.37467, *df* = 1, *P* = 0.540). The abundance of fungal endophytes in autumn was higher than summer and winter (*P* = 0.004 and *P* < 0.001, respectively) and the fungal abundance in spring was higher than winter (*P* < 0.001). White cabbage had a higher abundance of endophytes than radish (*P* = 0.012). In addition, the abundance of endophytes in stems and leaves were higher than in roots (*P* = 0.022 and *P* = 0.008, respectively). Similarly, the abundance of fungal endophytes showed no difference between organic and conventional farms (*P* = 0.542) (Figures 3E–H).

Season (χ^2^ = 9.9615, *df* = 3, *P* = 0.019) and plant tissue (χ^2^ = 18.789, *df* = 2, *P* < 0.001) were drivers to the species richness of fungal endophyte communities, while host plant identity and pesticide use were not (χ^2^ = 2.3535, *df* = 2, *P* = 0.308; χ^2^ = 0.020, *df* = 1, *P* = 0.887, respectively). Species richness was high in spring compared to summer (*P* = 0.039). Stem and leaf tissues of plants had significantly higher richness of endophyte species than roots (*P* = 0.003 and *P* < 0.001, respectively). No differences in endophyte species richness were observed among crop species or between organic and conventional farms (Figures 3I–L).

### Association of Different Land Use Proportions in the Landscape, Elevation Gradients, Key Local Factors and Seasons With the Fungal Endophytes

**FIGURE 4:**
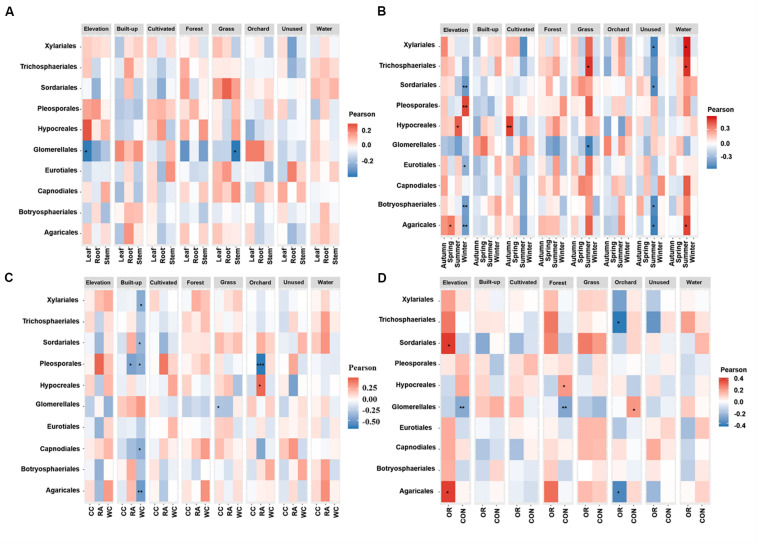
Associations between fungal endophyte abundance and elevation and numerical land-use variables based on correlation at levels of **(A)** plant tissue, **(B)** season, **(C)** plant identity, and **(D)** pesticide use. The abundance of each order of fungal endophyte is correlated with each of the environmental variables. Magnitude of the red color indicate the positive associations and magnitude of the blue color show the strength of negative association. “*” represents the significant correlation when *P* < 0.05, “**” represents the significant correlation when *P* < 0.01 and “***” represents the significant correlation when *P* < 0.001. CC, Chinese cabbage; WC, white cabbage; RA, radish; OR, organic farm; CON, conventional farm.

**FIGURE 5:**
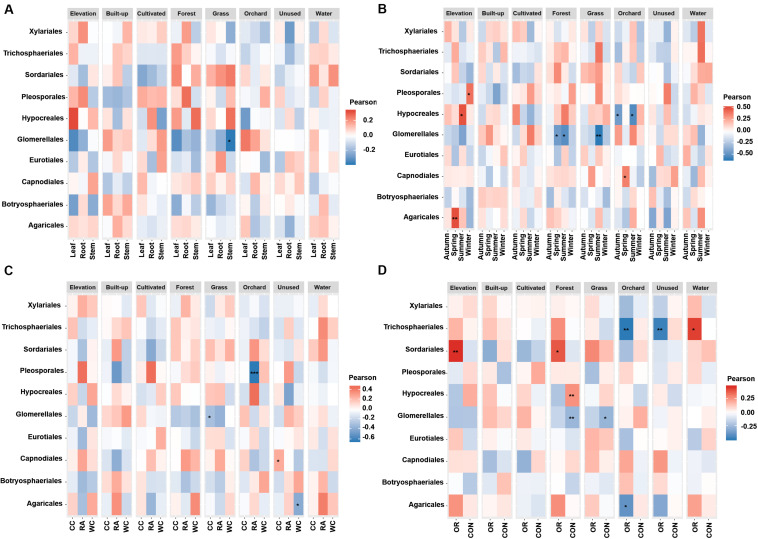
Associations between fungal endophyte species richness and elevation and numerical land-use variables based on correlation at levels of **(A)** plant tissue, **(B)** season, **(C)** plant identity, **(D)** pesticide use. The species richness of each order of fungal endophyte is correlated with each of the environmental variables. Magnitude of the red color indicate the positive associations and magnitude of the blue color show the strength of negative association. “*” represents the significant correlation when *P* < 0.05, “**” represents the significant correlation when *P* < 0.01 and “***” represents the significant correlation when *P* < 0.001. CC, Chinese cabbage; WC, white cabbage; RA, radish; OR, organic farm; CON, conventional farm.

Figures 4, 5 show the associations of species richness and abundance of the top 10 fungal orders with environmental variables based on the degree of correlation. The abundance of Glomerellales showed highly negative correlations with the elevation and proportion of grassland. The abundance of most endophytes showed a positive correlation with elevation in autumn, for white cabbage and organic farms, except Glomerellales with a negative correlation (Figures 4B–C). The abundance of most fungal endophytes had highly negative correlations with elevation and winter, except Pleosporales which exhibited a positive correlation (Figure 4B). Opposingly, elevation together with other growing seasons except winter had strong positive correlations with the abundance of Hypocreales and Agaricales (Figure 4B). The proportion of grassland in the landscape together with all local factors and different seasons was positively correlated with the abundance of Sordariales, Pleosporales and Hypocreales (Figure 4). The proportion of grassland in summer, for Chinese cabbage, on conventional and organic farms had positive correlations with the abundance of most endophyte orders, except Glomerellales which showed a negative correlation (Figures 4B–D). The proportion of cultivated land in the landscape during the autumn season had highly significant numbers of Hypocreales. Interestingly, the proportion of water bodies in the landscape during the summer season showed a strong positive correlation with the abundance of most endophytes, except Glomerellales which showed a negative correlation (Figure 4B). Conversely, the proportion of unused land in the landscape during the summer season showed a strong negative correlation with the abundance of most endophytes, except Pleosporales, Hypocreales and Glomerellales (Figure 4B). The proportion of forest in the landscape in conventionally managed fields had a significant positive correlation with the Hypocreales and a significant negative correlation with the Glomerellales (Figure 4D).

The species richness of Glomerellales had significantly negative correlations with the interaction between elevation, proportion of forest and grassland in the landscape with all local factors (including pesticide use, plant identity and plant tissue) across different seasons. In contrast, the species richness of Sordariales had positive correlations with the elevation, proportion of forest and grassland together with all local factors and different season (Figure 5). Elevation had a significant positive correlation with the species richness of Pleosporales during winter, Hypocreales during summer and Agaricales during spring (Figure 5B). Proportion of orchards in the landscape had significantly positive effects on species richness of Capnodiales during summer (Figure 5B). In contrast, the proportion of orchards in the landscape showed a significantly negative correlation with the species richness of Hypocreales during autumn and summer (Figure 5B), Pleosporales in radish crops (Figure 5C), and Trichosphaeriales as well as Agaricales in organically managed fields (Figure 5D).

The final CCA models explained 35% of the total variability in the assemblages of species richness in endophyte orders and 21% of the total variability in abundance. The first five, out of 10 orders, constrained eigenvalues (including season, plant tissue, plant identity, elevation and the proportion of grassland in the landscape), cumulatively explained 90% of the total variability in the assemblages of species richness in fungal orders and 92% in the abundance of top 10 endophyte orders (Supplementary Figure S2). Conversely, only small fractions of variability in terms of both species richness and abundance of fungal endophytes were explained by the pesticide use and the proportions of other land uses (cultivated land, forest, unused land and water).

After 999 permutations, CCA models showed that environmental predictors significantly contributed to structuring the fungal endophyte community patterns in terms of both species richness and abundance of orders (χ^2^ = 0.208, *P* = 0.001; χ^2^ = 0.263, *P* = 0.001, respectively) (Table 1). Additionally, during the permutation tests, season and elevation were found to be strong predictors to determine the assemblage structure of abundance (χ^2^ = 0.098, *P* = 0.001; χ^2^ = 0.016, *P* = 0.002, respectively) and species richness (χ^2^ = 0.076, *P* = 0.001; χ^2^ = 0.016, *P* = 0.015, respectively). Likewise, plant tissue and plant identity significantly contributed at the local scale to structuring the patterns of abundance (χ^2^ = 0.097, *P* = 0.001; χ^2^ = 0.017, *P* = 0.038, respectively) and species richness (χ^2^ = 0.070, *P* = 0.001; χ^2^ = 0.018, *P* = 0.021, respectively) in the endophyte orders. Considering the proportions of different land uses in the landscape, we found only grassland significantly determined endophyte abundance (χ^2^ = 0.013, *P* = 0.005). Conversely, no other land uses nor pesticide uses significantly contributed to structuring endophytes species richness and abundance (Table 1).

**TABLE 1:** Permutation tests for canonical correspondence analysis (CCA) of fungal endophyte abundance and richness in different plant tissues (root, stem and leaf) of the different plant identities (radish, white cabbage and Chinese cabbage) during different seasons (spring, summer, autumn and winter), under different pesticide uses (organic and conventional farm) in sites with varying proportions of different land-uses.

**Factors**	**Abundance**		**Richness**	
	**χ^2^**	***P* (>F)**	**χ^2^**	***P* (>F)**
Plant identity	0.017	0.038*	0.018	0.021*
Season	0.098	0.001***	0.076	0.001***
Plant tissue	0.097	0.001***	0.070	0.001***
Pesticide use	0.005	0.534	0.005	0.376
Elevation	0.016	0.002**	0.016	0.015*
**Land use**				
Forest	0.004	0.632	0.007	0.177
Cultivated	0.004	0.601	0.005	0.417
Grass	0.013	0.005**	0.009	0.062
Unused	0.005	0.449	0.004	0.671
Water	0.004	0.660	0.003	0.852
CCA model	0.263	0.001***	0.208	0.001***

The significance of constraint variables was tested by performing 999 permutation tests; “*” represents the significant constraints when P < 0.05, “**” represents the significant constraints when P < 0.01 and “***” represents the significant constraints when P < 0.001.

**FIGURE 6:**
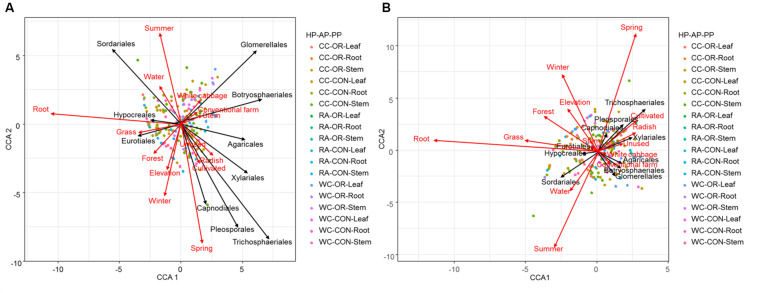
CCA ordination diagram with type II scaling represents the association of **(A)** abundance and **(B)** richness of top 10 fungal endophyte orders found in different plant tissues (root, stem or leaf) of different plant identities (radish, white cabbage or Chinese cabbage) during different seasons (spring, summer, autumn, or winter) under different pesticide uses (organic or conventional farm) across varying proportions of land-use variables. The arrow length and direction represent the magnitude of variance that can be explained by the explanatory and response variables. The perpendicular distance between fungal endophyte orders and explanatory variables reflects their correlations (<90° = positive correlation and >90° = negative correlation). The smaller the perpendicular distance, the stronger the correlation. Dots present a pooled plant tissue with different plant identity and pesticide use. HP, plant identity; AP, pesticide use; PP, plant tissue; CC, Chinese cabbage; WC, white cabbage; RA, radish; OR, organic farm; CON, conventional farm.

CCA ordination plots (Figure 6) highlighted the degree of associations between environmental predictors and fungal endophytes accounting the species richness and abundance. These plots clearly indicated that spring and winter versus summer, root versus stem, and white cabbage versus radish, were dissimilar in terms of species richness and abundance of endophyte orders. Intuitively, proportions of forest and grassland versus unused and cultivated land strongly opposed each other to promote endophyte species richness and abundance (Figure 6). Radish, cultivated land and spring showed strong positive associations with the abundance and species richness of Capnodiales, Pleosporales, Trichosphaeriales and Xylariales (Figure 6). White cabbage and conventional practices had strong positive associations with the abundance and species richness of Botryosphaeriales and Glomerellales. Moreover, root tissue of cruciferous crops and the proportion of grassland in the landscape were dominated by the abundance and species richness of Hypocreales and Eurotiales (Figure 6).

## Discussion

The present results illustrate the relative strength of, and interactions among, factors driving communities of endophytic fungi. This is of major practical significance. Among the fungi detected in the present study, some were previously found to be pathogens of plants or insects so merit further attention to exploit more fully for pest management.

Seasonal variation in the assemblage of fungal endophytes is demonstrated to be strong for cruciferous crops. Maximal colonization rate and abundance were observed during autumn, while the lowest was observed during winter. The abundance and species richness in spring were higher than that in summer. This may be due to temperature in autumn being suitable for fungal growth, while the lower temperature in winter reduce growth or colonization. In addition, farmers plant few cruciferous vegetables during summer, which might also explain the lowest diversity. Other studies found that the fungal endophyte abundance in *Pinus tabulaeformis* (Guo et al., 2008) and *Heterosmilax japonica* (Gao et al., 2005) were more abundant in spring than summer.

Proximate factors, host plant identity and plant tissue are also shown to be significant drivers of endophyte assemblage patterns. The selection of non-mycorrhizal cruciferous vegetables could avoid confounding effect of mycorrhizae and the selection of healthy plants could avoid the negative influence caused by the infection of pathogen (Lebreton et al., 2019). The results show that endophyte colonization rate and species abundance in cruciferous plants varied with plant identity, with the highest colonization rate and abundance observed in white cabbage and the lowest in radish. Radish had the lowest fungal colonization rate (35%) and species abundance (16) compared to white cabbage and Chinese cabbage (53.2% and 24.3, 42.6% and 18.8 respectively). A possible explanation is that different host plant species release different compounds into the surrounding soil, which can alter the properties of plant rhizosphere in favor of or against the colonization and uptake of endophyte fungi. In addition, the root system of different plant identities may act differently to allow fungal endophytes to colonize, which can range from deleterious to beneficial plant-microbe interaction. For example, the radish’s fleshy root system and its antimicrobial properties like acetone suppressed the colonization rate and diversity of endophytes (Beevi et al., 2009). Steinwender et al. (2015) found that *Metarhizium brunneum* was the most common species isolated from the roots of cabbage species (*Brassica oleracea*). However, in our study, we isolated 8 *Metarhizium* isolates from Chinese cabbage or radish but not from white cabbage roots. This difference may be due to the isolating methods used such as selective media (SDA versus PDA) or geographic variation. Using high-throughput DNA sequencing, Lebreton et al. (2019) found that Ascomycota were the dominant fungi in healthy Chinese cabbage roots, which is in accordance with our study, although we used culture-dependent methodology.

Larger scale factors including pesticide use patterns, landscape structure and elevation did have minor effects on determining the community structure of fungal endophytes. In the present study, farming practices showed no influence on the fungal endophytic community. In a study of the phyllosphere microbiota in grapevine, the richness and diversity of the fungal population were only minimally affected by chemicals and biological pesticides (Perazzolli et al., 2014). Similarly, fungicide and herbicide had no negative impact on the soil-borne entomopathogenic fungi. Several studies showed that entomopathogenic fungi in organic farms showed no difference with conventional farms (Goble et al., 2010; Tkaczuk et al., 2014). However, the root endophytic fungal rate in organic vineyards was significantly higher than conventional vineyards (Radić et al., 2014). Clifton et al. (2015) found that soil-borne entomopathogenic fungi in organic farms and accompanying margins were significantly higher than those of conventional farms. Although most studies showed that organic farming increases species richness, 16% of the studies showed opposite trend (Bengtsson et al., 2005).

The composition of the surrounding landscape differently influences the diversity patterns of local communities in an ecosystem (Pell et al., 2010; Tscharntke et al., 2012; Peay et al., 2013). The communities of fungal endophytes in organic farms were similar to those in natural grassland and dissimilar to their conventional farm counterparts (Verbruggen et al., 2010). The spores and species of arbuscular mycorrhizal fungi were strongly associated with grassland patches, followed by low- and moderate arable lands and the lowest in the high-input maize monocropping (Oehl et al., 2003). In another study of soil microbial communities, Eurotiales were abundant in grassland soils and Glomerellales in wheat fields (Manoharan et al., 2017). Similarly, we found that Eurotiales were strongly correlated with grassland and stems, and that Glomerellales negatively correlated with semi-natural habitats but positively associated with cultivated land in the landscape. In our study, forest did not show significant influence on the endophytic fungal distribution patterns. Similarly, the airborne fungal community showed no difference between forest and vineyard, indicating that habitat patches were not the main drivers of foliar fungal communities (Fort et al., 2016). In a study of symbiotic relationships, high water availability had no influence on the performance of endophytic-infected plants and disinfected plants (Kannadan and Rudgers, 2008). However, low water availability contributed 17% yield improvements of endophytic-infected plants than disinfected plants. In our results, waterbodies in the landscape showed a strong positive impact on the abundance of several fungal orders only in summer, which may be because the summer in the study region has a high temperature. The presence of water could improve the regional humidity, which can facilitate the symbiont relationships in a harsh environment.

Elevation significantly affected the species richness and abundance of the fungal endophytes. In a study assessing the richness of endophytic fungi along an elevation gradient in Costa Rica, Glomerellales increased in abundance with elevation increase, while Xylariales showed an opposite trend (Rojas-Jimenez et al., 2016). However, in our study, we found that elevation was negatively associated with the abundance of Glomerellales, while Xylariales was positively associated with the high elevation.

Dissanayake et al. (2018) compared the fungal endophytes using culture-dependent and culture-independent molecular approaches in grapevine (*Vitis vinifera*) stems and found an overlap of 53% of the fungal genera. In addition, OTUs (even with the highly abundant ones) could only be assigned to order, family or genus level. For a comprehensive understanding of the endophytic fungal community composition, both culture-dependent and culture-independent molecular approaches should be applied. Moreover, increasing the amount of each plant tissue could contribute to the improvement of accuracy of fungal community composition in plant tissues.

## Conclusion

Season, plant identity and plant tissue strongly affected endophytes community structure. However, conventional management practices significantly suppressed the belowground communities of endophytes but not aboveground communities. Elevation and grassland proportion in landscapes influenced endophytes. The interactions between season and local management with elevation and landscape factors had impacts on specific fungal orders. The existence of endophytes in crucifers growing system could contribute to the plant growth promotion and protection from herbivores. Our result suggests scope for farmers to promote the colonization and diversity of fungal endophytes in their cruciferous crops by managing the habitat around fields to retain and promote grassland and grow a diversity of cruciferous crop species (Arnold, 2007; Tscharntke et al., 2008; Jia et al., 2016). These generic guidelines merit further investigate to determine the extent of translation to protect crops from biotic threats.

## Data Availability Statement

The datasets generated for this study can be found in the National Center for Biotechnology Information (NCBI) database under accession numbers from MN202660 to MN202769.

## Author Contributions

JC, KA, LW, MY, and GG conceived and designed the experiments. JC, HS, XW, and KA performed the field sampling. JC, XW, FY, XX, MG, and KA carried out the isolation and identification of endophyte. JC, HS, and KA analyzed and interpreted the data. JC, HS, FY, and KA prepared figures and tables. JC, KA, HS, MG, and GG wrote the manuscript. KA, LW, MY, and GG supervised JC in the whole process. All authors discussed the results and reviewed the manuscript.

## Conflict of Interest

The authors declare that the research was conducted in the absence of any commercial or financial relationships that could be construed as a potential conflict of interest.

## Footnotes

1 https://www.ncbi.nlm.nih.gov/

2 https://submit.ncbi.nlm.nih.gov/subs/?search=SUB6020890
