# Supplementary material for: Fungal Endophyte Communities of Crucifer Crops Are Seasonally Dynamic and Structured by Plant Identity, Plant Tissue and Environmental Factors
Source: Front Microbiol. 2020 Jul 15;11:1519. doi: 10.3389/fmicb.2020.01519 (PMC7373767; doi:10.3389/fmicb.2020.01519)
Supplement: Supplementary file 1 [file Image_1.PDF]

Supplementary figures

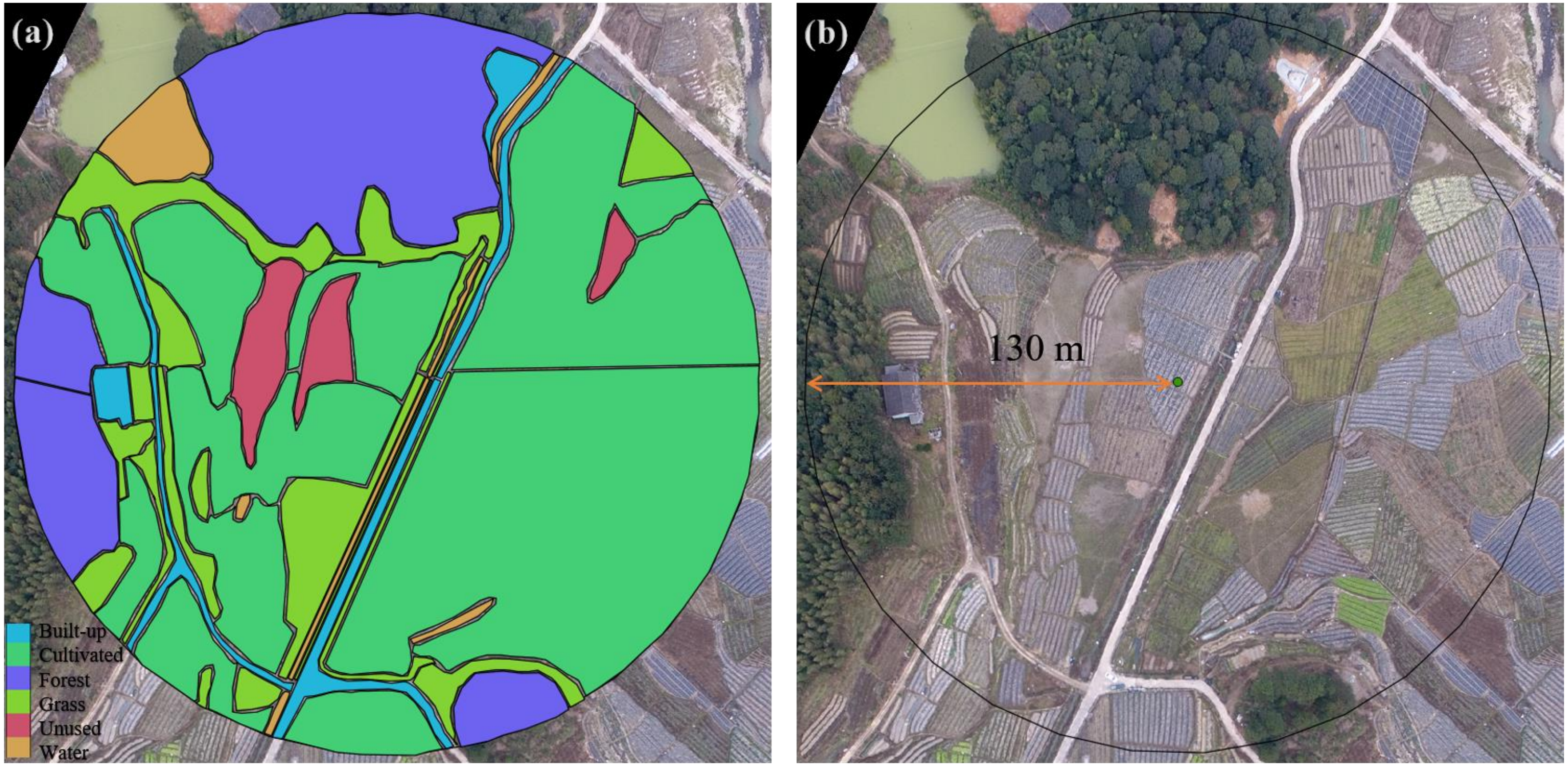

Supplementary Figure 1. (a) Mapping of georeferenced (using QGIS) and **(b)** drone-based high-resolution picture of a vegetable field in Fujian province, China

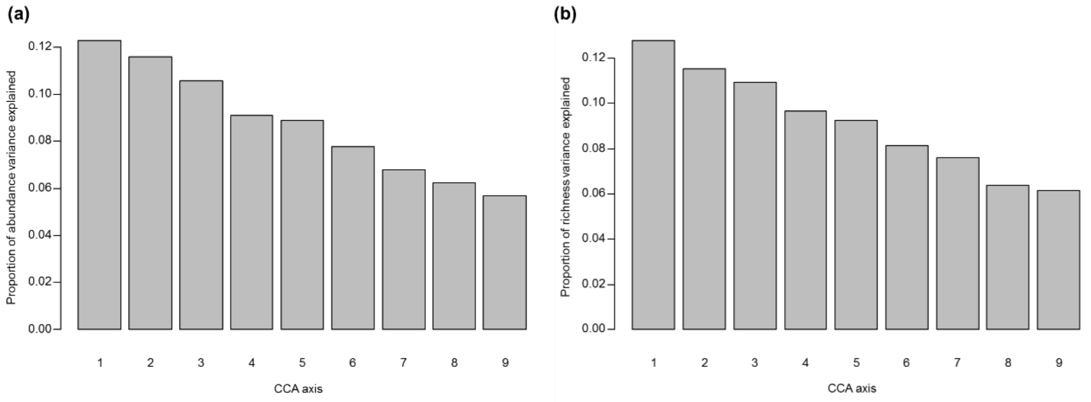

Supplementary Figure 2. Barplots of the proportion of variance in fungal endophyte (a) abundance and (b) richness, explained by each of the CCA axes
